# Perceptions of Abortion and Sexual and Reproductive Health in Chilean Medical and Midwifery Education: Protocol for a Mixed Methods Study

**DOI:** 10.2196/81427

**Published:** 2026-03-06

**Authors:** Lidia Casas-Becerra, Alejandra Ramm, Beatriz Pérez, Heidy Kaune, Mercedes Carrasco-Portiño, Adela Montero, Cecilia Bustos-Ibarra, Henry Castro-Arias, Claudio López-Labarca, Leonardo Reyes-Torres

**Affiliations:** 1 Law School Universidad Diego Portales Santiago, Metropolitana Chile; 2 Escuela de Sociologia Facultad de Ciencias Sociales Universidad de Valparaíso Valparaíso Chile; 3 Departamento de Psicología Universidad de la Frontera Temuco Chile; 4 Programa de Ética y Políticas Públicas en Reproducción Humana Facultad de Medicina Universidad Diego Portales Santiago Chile; 5 Centro de Investigación Biomédica Facultad de Medicina Universidad Diego Portales Santiago Chile; 6 Department of Obstetrics and Childcare School of Medicine Universidad de Concepción Concepción Chile; 7 Center for Reproductive Medicine and Integral Development of Adolescence Faculty of Medicine Universidad de Chile Santiago Chile; 8 Department of Social Work Faculty of Social Sciences Universidad de Concepción Concepción Chile; 9 Department of Obstetrics and Gynecology School of Medicine Universidad de Concepción Concepción Chile; 10 Department of Obstetrics and Childcare Universidad de Atacama Copiapó Chile

**Keywords:** ethics, public health, medical education, reproductive health, health services accessibility

## Abstract

**Background:**

University education for physicians and midwives in sexual and reproductive health (SRH), particularly regarding abortion, is shaped by broader societal debates, which are often polarized. Teaching at faith-based universities might limit the scope and quality of SRH education. The study is contextualized within Chile’s shifting legal and social landscape following the landmark 2017 reform under President Michelle Bachelet. This reform partially lifted Chile’s absolute ban on abortion, permitting it under 3 circumstances: risk to the mother’s life, fatal fetal anomaly, and a pregnancy resulting from rape. The protocol presented here intends to examine how the enacted legislation plays out in the curricula and how abortion is taught in secular and faith-based universities, which often take conflicting stances on abortion.

**Objective:**

This study aims to improve our understanding of how SRH, particularly abortion, is being approached by both students and faculty of medicine and midwifery in Chile. Specifically, it provides comparative evidence on attitudes, perceptions, and experiences related to abortion among medical and midwifery students and faculty in Chile.

**Methods:**

The study uses a cross-sectional and mixed methods design, with the participation of students and faculty within Chilean medical and midwifery programs. An interdisciplinary team developed and validated 2 instruments through an iterative process: a quantitative survey for undergraduate students to assess knowledge, attitudes, and competencies regarding abortion, and a semistructured interview guide for faculty to explore curricular decision-making. The instruments underwent expert content validation and pilot testing to ensure relevance to the local legal and educational context. Quantitative data will be analyzed using descriptive and inferential statistics, while qualitative findings will undergo thematic analysis to triangulate the current state of abortion training.

**Results:**

The funding for this study was awarded by the Diego Portales University (CG 1110689019), November 2022. Data collection for both the quantitative and qualitative components was completed as of July 2025. Recruitment finalized with 2795 students for the online survey and 57 faculty members for the semistructured interviews. Data analysis is in progress, and we expect to publish the findings from June 2026 onwards.

**Conclusions:**

This protocol examines the intersection of law, education, and public health by evaluating abortion training for Chilean medical and midwifery students. Transcending ideological debates to prioritize professional competency and health equity, the findings will guide educators and policymakers in preparing technically and ethically competent health care professionals.

**International Registered Report Identifier (IRRID):**

DERR1-10.2196/81427

## Introduction

Abortion rights have long dominated the agenda of Chilean feminist organizations. In 2017, under the leadership of then-President Michelle Bachelet, abortion laws evolved from a blanket ban to partial decriminalization [1]. Law 21.030 allows abortion when a woman’s life is at risk, when the fetus is fatally impaired, and when pregnancy is the result of rape [2]. As expected, the proposition to allow abortion on these grounds was fiercely resisted by conservative, religious, and right-wing groups. Chilean universities and academics became strongly engaged in the debate. While many scholars from non–faith-based universities argued in favor of the proposed legislation, their peers from faith-based universities argued forcefully against it, exposing deep fractures between faith-based and secular universities [1].

To be sure, Catholic opposition was led not by clergy but by the president of the Pontifical Catholic University, a trained physician who argued that institutions, including private universities, clinics and hospitals, should also be allowed to claim conscientious objector status [1,3,4]. Once the new legislation was passed, in a bid to prevent its enactment, conservative groups asked the Constitutional Court to review its constitutionality [5]. The Court ruled to confirm, but agreed to allow conscientious objector status to be extended to institutions, and to the rest of health care professionals, including technicians, who work in the surgical ward during the procedure. Faith-based universities operating clinics and teaching hospitals swiftly moved to do so.

A majority of Chileans do approve of abortion under certain circumstances, with surveys confirming widespread support, especially on the currently allowed 3 grounds [6,7]. A recent survey conducted by the respected Centre for Public Studies shows that support for abortion is actually growing. Indeed, asked in 1999 if “Women should always be able to choose an abortion,” 10% of respondents agreed; asked again in 2024, 34% agreed [7]. Likewise, support for abortion under “special circumstances” rose from 35% in 1999 to 50% in 2024. In other words, about 84% of survey respondents supported abortion in certain cases. Agreement with the view that “Abortion should always be banned” slipped from 55% in 1999 to 15% in 2024. Young people aged 18 to 24 years were especially supportive. In 2024, 45% agreed that “Women should always be able to choose an abortion” (in contrast to 27% agreement among respondents aged 55 years and older). Respondents with higher education also showed greater support for voluntary pregnancy termination [7].

Structural reforms spearheaded by the Pinochet dictatorship (1973-1990) laid the foundation for significant private involvement in providing public services such as retirement pensions, health care, and education. At the time, institutions of higher education numbered eight, 6 private and 2 public [8], all of which received State funding and charged no tuition. Along with promoting private involvement in higher education, the Pinochet regime also sought to cut public spending by transforming the 14 regional campuses of the large 2 State universities into as many freestanding universities. Today, both private and State universities are funded mostly through tuition fees. Since tuition is expensive, subsequent governments augmented funding support, and Bachelet introduced in 2018 free tuition for lower-income students.

Ministry of Education data indicates that in 2024, there were 58 universities, including 12 public and 46 privately-owned. At present, private universities account for more than 70% of undergraduate enrollment. Most universities are secular (44), while 14 are faith-based (all Catholic except for 1 Adventist) [9]. Universities' core values and mission statements might limit or veto curricular contents while shaping training delivery in particular ways. This has specific implications for the medical and midwifery professions, and more generally for the integration of young practitioners into the labor market and for the proper implementation of laws and public policy that liberalize abortion or guarantee health services.

Previous research surveyed the views and attitudes of medical and midwifery students and faculty members at the time of enactment of Law 21.030 across universities based in the Metropolitan region, where the capital city of Santiago is located [10-15]. Although the Metropolitan region is where most universities are based, previous research did not account for subnational differences. Findings revealed that the core values, whether secular or faith-based, of universities shape the content, methodology, and scope of teaching. Likewise, another study also showed that students’ attitudes toward sexual and reproductive health (SRH) differ according to their home regions. A multicenter study of SRH literacy in Chile found that students of medicine and health sciences have higher levels of knowledge compared to students from social sciences programs, but this varies by region [16]. Specific educational approaches further shape the attitudes and views of future health care practitioners. Against this backdrop, our study intends to delve into the attitudes, perceptions and experiences of Chile’s medical and midwifery students and faculty members regarding the SRH training they receive or deliver, with a special focus on abortion.

The role of universities in medical education and in the provision of abortion services has been studied in different settings, both in more liberal contexts, such as the United Kingdom [17], Germany [18], Canada [19], and Australia [20], and in more restrictive contexts, such as Brazil and Chile [21,22], with a focus on stigma, conscientious objection (CO), and the availability of services. In the United States, some studies share a concern about the impact of the Supreme Court’s decision in Dobbs (2022) on medical training and access to abortion services [23,24].

These studies reveal significant shortcomings in the training of medical students regarding abortion and the care of those who require these health services. Although students may support laws permitting abortion, their training is often deficient. A survey of medical students in Germany showed strong support for existing legislation and the belief that the law should be further liberalized. Students also expressed willingness to receive training in the future; however, 71% reported dissatisfaction with the curriculum at their faculty, and 75.5% of upper-year students indicated that they had not received any clinical training in abortion care [18].

A literature review also examined the extent and quality of education received by midwifery and nursing students, who are often considered more supportive professionals in relation to women’s reproductive health. The review found that, regardless of the legal context, abortion training is not prioritized and is difficult to access due to institutional barriers [25].

In Chile, there are no regulatory guidelines for training in Medicine or Midwifery regarding abortion. The Association of Medical Faculties of Chilean universities establishes a graduate profile outlining the competencies students must acquire; yet, it does not specify content regarding SRH [26]. However, the National Examination of Medical Knowledge, which is mandatory for medical graduates, qualifying them to practice in public health services and apply for medical specialties, includes content on spontaneous and induced abortion, as well as family planning. Nevertheless, medical schools retain the discretion to decide whether to teach these contents [27].

In the case of Obstetrics, Article 117 of the Sanitary Code establishes that midwifery professionals are responsible for SRH care and for carrying out procedures derived from medical diagnosis and treatment [28].

The international context described above prompts us to ask about the current state of abortion-related education received by medical and midwifery students in Chile, 7 years after the implementation of the law that partially decriminalized abortion in the country. As universities train the health care practitioners of the future, there is a need to assess whether curricular changes have been implemented to address cultural resistance by practitioners and instructors who either explicitly or implicitly convey negative attitudes toward abortion and reproductive autonomy. After the law entered into force, Robledo et al [29] found that the Health Ministry had to update physicians on vacuum aspiration techniques not taught in medical schools. Much criticism has emerged ever since regarding the poor knowledge, training, and awareness of health care practitioners about the new law [30], all factors that exacerbate the stigma surrounding abortion [30,31]. Key barriers to access abortion services identified include pervasive CO claims, particularly in abortion due to rape, revealing important regional differences [32,33], religiousness, and the stigma associated with performing an abortion [34,35]. Additional challenges include structural public health issues, such as underfunding, accessibility for the population in rural communities, insufficient mental health personnel, and lack of information campaigns about the law [32], given the connection between sexuality, reproduction, and abortion [36,37].

In this context, this study seeks to gain a deeper understanding of the views, perceptions, attitudes, and experiences of medical and midwifery students and faculty at Chilean universities regarding SRH education, with a particular focus on abortion. Specifically, the study aims to: (1) examine how SRH, and abortion in particular, is taught in medical and midwifery programs, including faculty members’ approaches to teaching and both students’ and faculty members’ perceptions of the training received; (2) ascertain the views and attitudes of medical and midwifery students and faculty members toward SRH, particularly in relation to abortion; (3) examine the perceptions and attitudes of both faculty members and students regarding patient confidentiality, CO, and the reporting of both lawful and unlawful abortions; and (4) explore students and faculty willingness to undergo training on abortion procedures and their views on facilitating access to legal abortion services.

We hypothesize that cultural changes may influence overall favorable opinions toward abortion among students in both degree programs, and that students from faith-based universities may report more stigmatizing attitudes toward abortion, more favorable views of CO, greater acceptance of reporting women for unlawful abortion, and more limited technical training in abortion care compared to students in secular institutions. We hypothesize that midwifery students may exhibit higher levels of empathy toward women compared to medical students. In addition, we anticipate identifying regional disparities, whereby students may encounter educational barriers associated with local cultural conservatism or limited access to clinical training. By examining the relationship between faculty attitudes and students’ perceptions, this study aims to explore how hidden curricula may shape the willingness of future professionals to provide abortion care, with potential implications for the implementation of the law and the protection of reproductive rights in Chile.

This study aims to improve our understanding of how SRH, particularly abortion, is being approached by both students and faculty of medicine and midwifery in Chile. Specifically, it provides comparative evidence on attitudes, perceptions, and experiences related to abortion among medical and midwifery students and faculty in Chile. Furthermore, its findings will provide evidence-based insights relevant for curriculum development, faculty training, and institutional guidelines, with potential implications for strengthening abortion-related training and improving the implementation of lawful abortion services. Ultimately, this study seeks to contribute to a more equitable access to abortion services and quality care.

## Methods

### Convergent Mixed Methods Design

A convergent mixed methods design is applied [38], which aims to achieve an enhanced understanding of a research problem through comparing and merging quantitative and qualitative research. As Creswell asserts, quantitative and qualitative data “provide different insights, and their combination contributes to seeing the problem from multiple angles and multiple perspectives [...] their combination adds up to not only more data but also a more complete understanding of the problem” [38]. In this kind of design, data gathering and analysis are done separately—usually in parallel—and then merged. Comparison is made possible through addressing similar topics and questions through both types of research (quantitative and qualitative).

In the case of the current research, we defined 2 distinctive yet complementary populations, medical and midwifery students and faculty, and we aimed to identify their perceptions, experiences, and attitudes toward abortion. Data gathering instruments—online survey questionnaires for students and semistructured interviews for faculty—address the same topics and also some specific ones for each population. After both types of results are obtained—quantitative and qualitative—inferences and interpretations from them are brought together in a discussion, to make meta-inferences or conclusions. We will first report the quantitative results to provide a “big picture” of tendencies toward abortion among students of medicine and midwifery. This will be followed by the qualitative findings on the same issues, based on interviews with faculty members, which will offer more detailed, contextualized, and in-depth insights. We will then conduct a follow-up discussion comparing results from both populations, students and faculty, seeking points of convergence as well as divergence. From this comparison, we will draw broader inferences, conclusions, and interpretations, which will in turn be contrasted with the existing literature on the subject. In doing so, we, and other researchers, will be able to assess whether our conclusions provide a valuable explanation of the proposed research problem. It should be highlighted that pragmatism has been frequently linked to mixed methods research [39-41], and hence this research draws upon pragmatism, applying a “pragmatic approach” [42,43]. Table 1 shows the overall study workflow, the estimated duration of each phase, and the participants involved in each of them.

**Table 1:** Study phases, duration, and participants.

Study phases			Duration (months)		Experts and participants
**1. Preliminary phase**			8		Research team (11 academic experts in law, medicine, midwifery, sociology, and psychology)
	1.1 Quantitative: instrument development and content validity through expert judgement				
	1.2 Qualitative: development and expert review of the semistructured interview guide				
**2. Pilot study**			3		
	2.1 Quantitative: comprehension of instructions and preliminary psychometric evaluation			50 Chilean medical and midwifery students	
	2.2 Qualitative: comprehension and adjustment of the semistructured guide			5 Chilean academics in medicine and midwifery	
**3. Sampling phase**			12		
	3.1 Quantitative sample selection and recruitment			1788 Chilean medical and midwifery students	
	3.2 Qualitative sample selection and recruitment			52 Chilean academics in medicine and midwifery	
**4. Data analysis**			12		—^a^
	4.1 Quantitative: psychometric study of the instruments and multivariate data analysis				
	4.2 Qualitative: coding and deductive and inductive analysis				
**5. Dissemination of results**					
	5.1 Congresses and seminars in different regions of the country	24		11 research team members	
	5.2 Scientific manuscripts	24		11 research team members	
	5.3 Presentation to university authorities	12		11 research team members	

^a^Not applicable.

### Participant Selection and Recruitment

In accordance with the convergent mixed methods design, and in order to enable comparison between the 2 populations (students and faculty), participant selection criteria are similar for both the student survey and faculty semistructured interview. These criteria are (1) type of degree program (medicine or midwifery), (2) type of university (secular or religious), (3) geographical area (Northern, Central, Metropolitan, and Southern), and (4) sex (female and male). With regard to gender, it should be noted that students and faculty members who self-identify as lesbian, gay, bisexual, transgender, and queer or questioning, and others are not excluded from the study.

The planned quantitative study involves applying a multivariate correlational, cross-sectional, observational design to a nationwide sample of medical and midwifery students using a range of strategies. The study is designed to be conducted in each of the country's 3 macroregions: in the North, in one of its 5 regions; in the center, in one of its 3 regions; and in the South, in 3 of its 7 regions, because of its larger geographical area compared to the other macro-regions. In addition, the Metropolitan area will be considered independently, as it corresponds to a region within the Central zone where the nation's capital and the highest concentration of population and universities are located; to protect participant identity, specific regions will not be named.

In 2022, total enrollment at medical and midwifery schools was 30,862, distributed across Northern Chile (n=1686, 5.46%), Central Chile (n=10,972, 35.55%), Southern Chile (n=4138, 13.40%), and Metropolitan Santiago (n=14,066, 45.57%) [44]. Regions were treated as independent populations based on accessibility and enrollment in the target fields. For sample size calculation, a stratified sampling design by geographic region was used. Stratum-specific sample sizes were calculated to estimate proportions with a 95% confidence level and ±5% precision, assuming *P*=0.5 and applying finite population correction. Based on 2022 enrollment figures, the resulting target sample sizes were Metropolitan Santiago (*P*=14,066; n=375), Northern Chile (*P*=433; n=205), Central Chile 1 (*P*=2803; n=339), Central Chile 2 (*P*=4124; n=281), Southern Chile 1 (*P*=1497; n=307), and Southern Chile 2 (*P*=1034; n=281). The total sample size corresponds to the sum of stratum-specific targets (n=1788), with adjustment for anticipated nonresponse.

Once the target sample size was defined for each region, the distribution and selection of participants were conducted using a nonprobabilistic stratified sampling method, which consists of dividing the population into homogeneous subgroups (strata) and selecting participants by convenience within each one. This stratification is based on three key variables due to their correlation with attitudes toward abortion: (1) type of degree program, (2) type of university (secular or religious), and (3) gender. The sample was designed to include equal proportions of students from medicine (n=894, 50%) and midwifery (n=894, 50%), as well as from secular (n=894, 50%) and religious (n=894, 50%) universities, where both types of institutions were available. In some selected regions (one in the northern zone and one in the southern zone), there are no religious universities offering these programs; only secular universities were included in those areas. Gender stratification was applied within each field of study to reflect the national enrollment distribution (medicine: n=392, 43.8% men and n=502, 56.07% women; midwifery: n=40, 4.46% men and n=854, 95.53% women), while also ensuring adequate participation of men, given their typically lower response rate. The survey was distributed, and students were recruited through a variety of methods, notably (1) institutional contacts, (2) personal team contacts, and (3) social media invitations (Facebook [Meta], Instagram [Meta], and WhatsApp [Meta]).

Participant selection of faculty for the semistructured interviews followed the same criteria used to stratify the quantitative sample: type of degree program, type of university, geographical area, and gender. In accordance with the study’s pragmatic approach, the total number of faculty and their distribution across the sampling variables were determined based on considerations of information power, that is, the sufficiency of the number and diversity of cases [45], as well as practical considerations related to the feasibility of conducting the proposed interviews. A sample of 52 faculty was designed. Table 2 shows the distribution of the qualitative sample.

Of the total sample, half of the cases (n=26) are from the medical faculty and the remaining half (n=26) from the midwifery faculty. A similar distribution is planned in terms of gender, with equal numbers of women and men. Given the smaller number of religious universities compared to secular institutions, and the greater reluctance of faculty from religious universities to participate in interviews on SRH issues, particularly abortion, the sample is estimated to include 20 faculty members from religious universities and 32 from secular universities. Regarding subnational diversity, the number of cases in each geographical area is determined based on the number and type (secular or religious) of universities offering medical and midwifery training in each region. Accordingly, 16 interviews are allocated to the Metropolitan area and 16 to the Southern region, as both geographical areas have the highest concentration and diversity of universities nationwide. In contrast, 8 interviews are allocated to the Northern region, reflecting the lower number of universities and the absence of religious institutions. An intermediate number of 12 interviews is allocated to the Central region, given the limited presence of religious universities.

**Table 2:** Qualitative sample distribution^a^.

Geographical zone and gender		Confessional universities		Secular universities				Total
		Medicine	Midwifery	Medicine		Midwifery		
					
**Northern**								
	Female	0	0		2		2	4
	Male	0	0		2		2	4
**Central**								
	Female	1	1		2		2	6
	Male	1	1		2		2	6
**Metropolitan**								
	Female	2	2		2		2	8
	Male	2	2		2		2	8
**Southern**								
	Female	2	2		2		2	8
	Male	2	2		2		2	8
Total		10	10	16		16		52

^a^In the Northern area, there are no confessional universities with schools of medicine and/or midwifery. While in the Central zone, only one of its component regions has universities with schools of medicine and/or midwifery.

Recruitment is designed to proceed through formal and informal team contacts, and respondents will be asked to provide leads for additional interviews. As stated, it is anticipated that securing interviews with academic staff from faith-based universities will pose a challenge. Unless prospects prefer an in-person session, interviews will be conducted remotely on video conferencing platforms such as Zoom. Respondents will be asked to be in a location that guarantees privacy. Conducting interviews remotely is considered more appropriate due to participants’ time constraints. In addition to teaching duties, most medical and midwifery lecturers are health care providers and have little free time. Sessions are designed to last a maximum of 30 to 45 minutes.

### Data Collection Instruments

A student survey and a faculty interview guide were developed through a rigorous, iterative process carried out collaboratively by an interdisciplinary research team comprising law scholars, physicians, midwives, sociologists, and psychologists. All team members are academics with expertise in SRH, health law, bioethics, and/or medical education in Chile. Both the faculty interview guide and the student survey were developed based on previous empirical and theoretical research in the field [9,11-14]. The research team met on a weekly basis over an 8-month period to develop the student survey and the faculty interview guide.

In relation to the student survey, the research team defined the conceptual and theoretical framework underlying each construct, reviewed existing validated instruments, and adapted or developed items to ensure contextual relevance to the Chilean legal, educational, and health care settings. This process provided expert-based content validation, allowing for the refinement of item wording, response formats, and scale structure prior to pilot testing.

The student survey comprises a variety of instruments, including a questionnaire designed to collect sociodemographic data, such as age, gender, ethnicity, political and religious affiliation, church attendance, type of school attended, nationality, type of degree program, and type of university attended (public or private; secular or faith-based; region). Other instruments are listed in Table 3. Instruments using a 5-point Likert scale have response options consistently labeled, ranging from 1=Strongly disagree to 5=Strongly agree. Higher scores indicate a greater presence of the construct being measured.

**Table 3:** Student survey scales.

Factors and definition of constructs		Items	Scale and comments
**1. Scale on the Centrality of Political affinity in Self-Concept** [31]			
	Unidimensional. Prominence and subjective importance that participants attach to identification with a right-wing political orientation in the construction of their self-concept	3	5-point Likert scale. In addition, it presents two additional, independent items on the left and center political orientation
**2. Scale on Conscientious Objection to Abortion: Positioning and Reasons ad hoc**			
	4 factors. Factors 1, 2, and 3 capture the motivations for declaring CO^a^: (1) Value, ethical, or religious reasons; (2) fear of consequences; (3) professional reasons; and (4) intention to declare conscientious objection	17	5-point Likert scale. These factors can be combined to detect pseudo-CO, ie, the declaration of CO for reasons other than those legally supported: fear of consequences and professional reasons
**3.** **Conscientious Objection to Abortion Knowledge Scale ad hoc**			
	Unidimensional. Knowledge of the rights and obligations of CO to abortion, as required by Chilean law	4	3 response options: “Yes,” “No,” or “Don't know”
**4.** **Support for Individual and Institutional Conscientious Objection ad hoc**			
	Nonpsychometric. Support for the legality of individual and institutional CO and associated competencies	7	Various response scales
**5. Intentionality to Promote Abortion Law Compliance Scale ad hoc**			
	2 factors. Participants' intentionality to facilitate or hinder access to legalized abortion: (1) promote compliance with the law and (2) hinder compliance with the law	6	5-point Likert scale
**6.** **Abortion Attitudes Scale: Criminalization, Acceptance, and Entitlement ad hoc**			
	3 factors: (1) criminalization, beliefs about the conditions under which an abortion should be punishable by imprisonment; (2) acceptability, beliefs about the conditions under which an abortion is acceptable, and finally; and (3) right, belief in abortion as a right	19	5-point Likert scale
**7. Abortion Attitudes Scale: Reporting and Confidentiality in Professional Practice ad hoc**			
	3 factors: (1) willingness to report. This factor assesses the belief in the professional duty to report abortion situations not covered by the law; (2) risks associated with not reporting. This dimension measures beliefs about the risks of not reporting abortion situations not protected by law; and (3) willingness to maintain confidentiality. Preference for Confidentiality, based on the belief that professional secrecy should prevail over reporting in abortion situations not protected by law	20	5-point Likert scale
**8.** **Abortion Attitudes Scale: Best Friend Support ad hoc**			
	Unidimensional. Intention to help a woman with whom one is a friend who seeks an illegal abortion	7	5-point Likert scale
**9.** **School sex education and university training in SRH^a^ ad hoc**			
	Nonpsychometric. Type and quality of sexuality education received at school and university, and the opinion about university education on abortion issues	6	Various response scales

^a^CO: Conscientious Objection.

^a^SRH: sexual and reproductive health.

### Pilot Study

The pilot study helped to assess the comprehension of instructions and items and provided a preliminary psychometric evaluation of the instruments. After the exclusion of 5 participants due to incomplete responses, the final sample consisted of 50 students, exceeding recommended sample size guidelines for this type of study based on statistical considerations and decision-making requirements for early-stage instrument development [46,47].

Of the total participants, 66% (33/50) were women. Overall, 54% (27/50) were enrolled in medicine, while the remaining 46% (23/50) were studying midwifery. Regarding level of training, most participants were in advanced years of their programs (first to fourth year: 20/50, 40%; fifth to seventh year: 30/50, 60%). In addition, 36% (18/50) were enrolled in universities with a religious orientation. The sample also included representation from all macro-regions of the country (Northern: 5/50, 9.1%; Central: 22/50, 40%; Metropolitan: 12/50, 21.8%; Southern: 11/50, 20%). Overall, this diversity contributed to meeting the representativeness recommendations proposed by Johanson and Brooks [47] for pilot studies.

Decisions regarding scale refinement were based on analyses of inter item correlations (bivariate correlations between items), internal consistency, and corrected item–total correlations, both for the full scales and for their theoretical factors. These criteria were applied systematically in order to identify redundant items or items showing weak associations with the overall scale, prioritizing parsimonious and conceptually coherent versions of the instruments. In addition, participants’ subjective evaluations of the clarity and comprehensibility of instructions and items were considered:

1. Scale on the Centrality of Political Affinity in Self-Concept (CPolA): the research team explored the possibility of expanding this previously published scale [48] by adding 6 items intended to measure the centrality of centrist and left-wing political orientations. However, analyses indicated conceptual redundancy. Consequently, only 2 additional items, one for each political orientation, were retained as a complement to the original scale.
2. Scale on Conscientious Objection to Abortion: Positioning and Reasons (COA-PR), ad hoc. Initially composed of 19 items, 1 item was removed due to low association, while another item corresponding to the theoretical factor “Intention to Object” was transferred to the SOC-Ind/Ins scale based on theoretical and statistical criteria. The final version of the COA-PR scale, therefore, consisted of 17 items.
3. Conscientious Objection to Abortion Knowledge Scale (Kno-COA), ad hoc. A difficulty was identified with the response format, leading to a change from a 5-point Likert-type scale to a trichotomous nominal response scale (yes, no, or don’t know). The number of items (four) and their wording remained unchanged.
4. Support for Individual and Institutional Conscientious Objection (SOC-Ind/Ins): this nonpsychometric scale, composed of 7 items, required wording modifications in 4 items.
5. Intentionality to Promote Abortion Law Compliance Scale (InC-AL). Of its 7 items, 1 was removed due to low association, and another required wording modification.
6. Abortion Attitudes Scale: Criminalization, Acceptance and Entitlement (AA-C/A/E). Of the original 23 items, 3 were removed due to low association (2 from the “Acceptance” dimension and one from the “Entitlement” dimension). In addition, 2 highly similar items within the “Acceptance” dimension showed high interitem correlation and were therefore merged into a single item. Finally, the wording of one item in the “Criminalization” dimension was modified.
7. Abortion Attitudes Scale: Reporting and Confidentiality in Professional Practice (AA-R/C). One item from the “Willingness to Report” dimension was removed due to low association, resulting in a final scale of 20 items. The wording of 2 additional items was modified.
8. Abortion Attitudes Scale: Best Friend Support. In this 7-item scale, only 2 items required wording modifications.
9. School Sex Education and University Training in SRH: this 6-item nonpsychometric scale did not undergo any modifications.

The faculty semistructured interview guide was designed to address the same topics as the student survey, which is central to a convergent mixed methods design. The first part of the interview guide delves into three key themes: (1) delivery of SRH education, with a focus on abortion; (2) ethical dilemmas associated with the ongoing criminalization of abortion beyond the permitted grounds; and (3) perceptions on the prevalence of abortion in Chile and on CO issues, while the second part involves a close-ended sociodemographic questionnaire designed to gather information on age, gender, level of education, political affinity, religious affiliation, years teaching SRH, and type of university where employed, among others.

The interview guide was tested through 5 pilot interviews involving 3 men and 2 women; 3 midwives and 2 physicians; 3 participants from secular universities and 2 from religious universities; and participants from different geographical areas (2 from the Central area, 1 from the Metropolitan area, and 1 from the Southern area). Testing of the interview guide resulted in the addition of one question regarding faculty members’ own experiences as students in relation to the SRH training they received, particularly concerning abortion, as well as improvements to question wording. The final semistructured interview guide is provided in Multimedia Appendix 1.

### Data Analysis

Two levels of analysis are considered for the students’ survey data. First, a large part of the results will be approached as theoretical constructs, as per classic test theory. This will help develop psychometric instruments with potential for replicability, comparison and guaranteed reliability and validity. This will also help categorize and differentiate participants in the resulting theoretical constructs. Results will then be approached item by item in order to discern the scope of observed variables in detail.

Analysis of the psychometric properties of designed scales involves several steps: (1) analysis of the discriminative ability of items: skewness, kurtosis, item-total correlation, and bivariate correlation will be checked; (2) study of the factorial structure by means of cross-validation, applying exploratory and confirmatory factor analysis in independent subsamples; (3) analysis of the internal consistency of participant scores. McDonald omega coefficient or KR20 will be used as appropriate; and (4) analysis of measurement validity based on links to other variables. Assuming parametric assumptions are met, Pearson correlations and mean differences between 2 or more groups (Student *t* test or one-factor ANOVA) will be used. For analysis of results as theoretical constructs or independent observed variables, various strategies will be used: (1) Descriptive statistics of central tendency and frequency; (2) Bivariate statistics, such as Pearson correlations; (3) Means comparison between 2 or more groups (student *t* test or one-factor ANOVA or MANOVA); and (4) Multivariate analysis techniques intended to understand the relative importance of various variables to predicting or explaining the phenomena under review, for example, multivariate multiple and binary logistic regression, factor ANOVA and multivariate analysis of variance, or structural equation modelling [48-51].

Qualitative coding is going to be carried out through “structural coding,” which consists of using the questions from the interview guide to create a priori codes [49]. As stated by Saldaña, structural coding is particularly appropriate for studies involving multiple participants and semi-structured interviews. Structuring coding allows large fragments of the interviews to be grouped into a small number of broad codes, which identify the main topics addressed in the interviews. In this way, it helps to initially categorize the data corpus, identifying similarities, differences and relationships among different interview transcripts. Thereafter, similarly coded segments -that have been coded together- are further analyzed and recorded, producing a more detailed coding scheme for each main topic [49]. Five structural codes were defined a priori: (1) teaching SRH and abortion (approach based on institutional values, teaching methodologies, academic freedom, and hidden curriculum), (2) knowledge of legal abortion protocols, (3) medical ethics, patient confidentiality and reporting of illegal abortions, (4) conscientious objector status, and (5) expanding abortion grounds and the right to choose. In turn, these predefined codes will be revised, modified, and expanded, involving inductive analysis too. ATLAS.ti v. 24.2.0 (Lumivero) qualitative data analysis software will be used to ensure systematic, consistent, and transparent analysis. Codes will also be analyzed per interviewee characteristics in order to identify differences according to variables such as type of university (faith-based or secular); field of endeavor (medicine or midwifery); gender, teaching experience, and geographical area, among others.

Drawing on the pragmatic approach endorsed by this research [43,52] and the established qualitative quality principles, the study will address credibility through systematic and iterative analysis, dependability through transparent documentation of analytic decisions, and reflexivity through critical examination of researchers’ assumptions. Likewise, transferability becomes a question about how the findings from a specific context might be meaningful to another. These criteria will be applied pragmatically, with an emphasis on ensuring that the qualitative findings are robust, interpretable, and meaningfully integrated with the quantitative results.

### Ethical Considerations

The research protocol was reviewed and approved by the research ethics committee of Diego Portales University (No 022-2022). Pursuant to Laws No 20.120 [53] and 19.628 [54], and article 10 of the Decree 114 of the Ministry of Health (November 19, 2011) [55], for a multicenter study, the ethics approval is required by a single accredited ethics committee on scientific research. The University of Concepción and the Austral University of Chile approved the feasibility of the study in their local communities. The ethics committees of La Frontera University, the Faculty of Medicine of the University of Valparaíso, the Faculty of Medicine of the University of Chile, and the University of Atacama waived approval, given the ethics authorization by the Diego Portales University research ethics committee.

All faculty participants signed an informed consent. For students, the informed consent was displayed electronically, and they had to consent before accessing the online survey. Informed consent forms provided information on research objectives and methods (questionnaires or interviews, as applicable), confidentiality of personal data, the voluntary nature of participation, the right to withdraw at any time, and the contact information of the investigator responsible for answering queries. During recruitment, analysis and results dissemination, all personal data will be treated as confidential. Codes have been used to protect the interviewee’s identity. As a token of appreciation for their participation, 70 gift cards equivalent to US $23 were raffled among all students who completed the survey. Multimedia Appendix 2 SPIRIT (Standard Protocol Items: Recommendations for Interventional Trials) 2025 checklist.

## Results

The funding for this study was awarded by the Diego Portales University (CG 1110689019) in November 2022. Data collection was completed in July 2025. As of July 2025, a total of 2795 medical and midwifery students were enrolled in the quantitative phase of the study and answered the online survey, and 57 faculty members were recruited for the qualitative stage and were interviewed. We are currently conducting the data analysis. The results are expected to be published from June 2026 onwards.

## Discussion

### Anticipated Findings

This study represents the first nationwide effort to comprehensively assess the interaction between institutional ideologies and SRH training in Chile following the 2017 legal reform. We anticipate that our findings will reveal a significant gap between the competencies required by Law 21.030 and the actual training provided by medical and midwifery schools.

Current literature on abortion education highlights a global deficit in medical training [17-22,25]. Shifting political contexts can lead to improved abortion training, or conversely restrict it, as appears to be occurring in the United States in the Supreme Court’s decision in Dobbs [23,24]. Studies in various countries reveal that even where there are liberal abortion laws, medical curricula often lack practical training and fail to address the social aspects of SRH in the UK [17] and Germany [18]. In addition, prior evidence suggests that training gaps may be more pronounced in faith-based or otherwise institutionally restrictive settings [56,57]. Our study will contribute to this body of work by examining a context of “partial decriminalization,” a scenario common in Latin America but less studied in terms of its educational impact. The results should also help provide an empirical basis for developing more effective teaching strategies adapted to the socio-cultural realities of students, thus contributing to educational policies that promote up-to-date, sound, and more inclusive SRH education.

A major strength of this study is its mixed methods design, which allows integration between quantitative (survey) and qualitative data (interviews). The inclusion of both medicine and midwifery students is key, as midwives in Chile play a central role in women’s health, providing most of the health care throughout their life course and often holding more progressive views on SRH than physicians. Additionally, the national scope will allow us to enrich the analysis by understanding the diverse realities of teaching across the country and reducing the bias of concentrating solely on the capital city.

However, our study has limitations given the still-controversial nature of abortion in Chilean society. The primary challenge is the potential self-selection bias; students and faculty with strong pro- or antiabortion views may be more willing to participate than those with neutral positions. Furthermore, recruitment of faculty from conservative faith-based institutions was a significant challenge due to fear of institutional reprisal or stigma. To mitigate this, we implemented strict anonymity protocols and used a recruiting strategy through reliable professional networks to build trust with possible participants. Finally, the cross-sectional nature of this study captures a specific moment in time, and longitudinal studies would be needed to assess how educational interventions could change attitudes over the long term.

The evidence generated by this study will provide guidance to faculty members seeking to address controversial issues in the classroom and will help to define minimum curricular standards for SRH training in Chile. The study should also help provide an empirical basis for developing more effective teaching strategies adapted to the sociocultural realities of students, thus contributing to educational policies that promote up-to-date and more inclusive SRH education. If our hypothesis regarding the training gap is confirmed, the results will support the need for the Ministry of Health and the Ministry of Education to collaborate on standardized guidelines that ensure all health graduates possess the competencies to comply with the law, regardless of the university where they obtained their degree. Future studies should explore the effectiveness of specific educational interventions in reducing abortion stigma among students.

To maximize impact, findings will be disseminated through peer-reviewed publications in journals focused on medical education, public health, and reproductive rights. We plan to present results at national medical and midwifery conferences to directly reach educators. Additionally, policy briefs will be developed for university deans and government officials, highlighting recommendations to improve SRH curricula.

### Conclusions

This research protocol addresses a critical intersection between law, education, and public health. By evaluating how future physicians and midwives are taught about abortion in a changing legal frame, the study will surpass ideological debates to focus on professional competency and promote health care equality for women and pregnant people. The findings will provide a route for educators and policymakers to ensure that the next generation of Chilean health care providers is technically competent and ethically prepared to fulfil the needs of a diverse population.

## Appendix

Multimedia Appendix 1 Interview guide.

Multimedia Appendix 2 SPIRIT 2025 checklist.

## Abbreviations

AA-C/A/E: Abortion Attitudes Scale: Criminalization, Acceptance and Entitlement
AA-R/C: Abortion Attitudes Scale: Reporting and Confidentiality in Professional Practice
CPolA: Scale on the Centrality of Political Affinity in Self-Concept
CO: conscientious objection
COA-PR: Scale on Conscientious Objection to Abortion: Positioning and Reasons
InC-AL: Intentionality to Promote Abortion Law Compliance Scale
Kno-COA: Conscientious Objection to Abortion Knowledge Scale
SOC-Ind/Ins: Support for Individual and Institutional Conscientious Objection
SPIRIT: Standard Protocol Items: Recommendations for Interventional Trials
SRH: sexual and reproductive health

## Data Availability

All data generated or analyzed during this study will be included in the published articles and their supplementary information files.
