# Supplementary material for: Perceptions of Abortion and Sexual and Reproductive Health in Chilean Medical and Midwifery Education: Protocol for a Mixed Methods Study
Source: JMIR Res Protoc. 2026 Mar 6;15:e81427. doi: 10.2196/81427 (PMC13005057; doi:10.2196/81427)
Supplement: Multimedia Appendix 1 [file resprot_v15i1e81427_app1.docx]

**Interview guide**

**TEACHING EXPERIENCE**

Q1. To begin with, in your experience, how are sexual and reproductive health topics taught in this degree programme?

i. And how are they taught at faculty and/or university level?

ii. And in your career, have you been involved in other academic centres or civil society groups?

Q2. What sexual and reproductive health content is covered in this degree programme?

Q3. Do you address professional secrecy and confidentiality? How do you do so?

Q4. In your teaching, do you address sexuality and reproduction in general, or do you also address the following specific groups?

i. adolescents

ii. sexual diversity

iii. immigrants (Haitians, Peruvians, Bolivians, etc.)

iv. other groups: people with HIV, mental disabilities, hereditary diseases, alcoholics and/or drug addicts, ethnic groups (Mapuche, etc.)

Q5. Specifically, regarding abortion what topics are covered or addressed?

i. Are ethical issues associated with abortion discussed?

ii. What about legal aspects associated with abortion?

iii. How is abortion in people under 18 addressed?

Q6. Following the decriminalisation of abortion on three grounds, in what ways, if any, have pedagogical approaches to sexual and reproductive health changed?

i. How were these topics taught at university before the enactment of the IVE law?

ii. What was your experience as a student when addressing this topic?

Q7. Abortion raises certain dilemmas in the practice of health professions. In this regard, how do you address the following issues in your teaching? (If the person does not teach directly, ask about teaching at the degree level)

i. How would you address in class the case of a woman seeking an abortion within the three grounds?

ii. What about the case of a woman who consults you about having an abortion outside the three grounds?

iii. In cases where a woman presents with post-abortion complications outside the three grounds or who is suspected of having had an abortion outside the three grounds, how do you address the conflict between professional secrecy and the duty to report with your students? (*ask if this change depending on whether the situation occurs in a public or private hospital *)

iv. How do you address the conscientious objection of health professionals versus a woman's right to receive sexual and reproductive health services in your teaching?

Q8. How do students respond when abortion-related topics are addressed during both theoretical and practical training (e.g., classes, clinical placements, etc.)?

i. Which specific topics elicit the greatest interest?

ii. Which topics pose the greatest challenges to address?

iii. How informed do you perceive students to be regarding the current reality of abortion in Chile?

iv. Do you believe students feel free to express their views on abortion?

Q9. Do you consider that graduates of this university have the necessary technical, ethical, and legal skills to provide abortion care, or do they need additional training?

Q10. Thinking about how the teaching of sexual and reproductive health ought to evolve in the future within this degree/university:

i. What curricular content do you believe should be incorporated?

ii. And regarding teaching and learning methodologies?

iii. And specifically, regarding abortion?" (if not mentioned)

Q11. In a broader context, how would you visualise the future evolution of sexual and reproductive health education in Chile?

Q12. To what extent do you feel free to teach sexual and reproductive health topics based on what you personally consider essential? (This question addresses academic freedom, probing for institutional directives without leading the response)

**PROFESSIONAL EXPERIENCE**

Q13. In your experience, how common is induced abortion?

Q14. Regarding abortion:

i. Under what circumstances do you believe a woman should have access to abortion?

ii. Which professionals should be permitted to perform an abortion? (Doctors, midwives, others)

Q15. Regarding conscientious objection:

i. What is your view on conscientious objection?

ii. Specifically: which health professionals, technicians, or workers should have the right to exercise it? (Nurses, midwives, doctors, pharmacists, administrative staff, others).

iii. In the current discussion regarding a new Constitution, a proposal has been made to include institutional conscientious objection, without limitations, for universities. What is your opinion on this proposal?"

Q16. Is there anything else you would like to add?

Stop recording. Request the following identification and socio-demographic data

1. Record sex of the interviewee: a) Female b) Male

2. Name:

3. Contact telephone number:

4. Contact email:

5. University(ies) and campus(es) where you currently lecture in sexual and reproductive health and/or ethics:

6. Age and total years teaching sexual and reproductive health:

7. Aside from teaching, do you practise clinically? If so, specify where (private clinic, private practise, public hospital, other):

8. Level of studies and specialisation (specifying institutions and countries where studies were undertaken):

9. Children (number):

10. In terms of political affinity, do you feel closer to? (mark, do not read)

Right-wing

Centre-right

Centre

Centre-left

Left-wing

11. In terms of beliefs and religion, do you consider yourself?

Practising Catholicism. Specify congregation/movement.

Non-practising Catholic.

Evangelical.

Atheist, agnostic, or non-believer.

Other belief or religion: specify.

Thank the participant and request cooperation to contact their students for an online survey on this same topic. state that a questionnaire will be sent via email to be completed with personal details.

• Interview duration:

• Interviewer:

• Location:

• In-person or other:
